# Strengths and limitations of *in vitro* and animal models to advance understanding of human diet‒microbiome interactions

**DOI:** 10.1080/29933935.2026.2636336

**Published:** 2026-03-03

**Authors:** Alexa R. Weingarden

**Affiliations:** a Division of Gastroenterology, Hepatology, & Nutrition, Department of Medicine, University of Minnesota Medical School, Minneapolis, MN, USA; b Center for Immunology, University of Minnesota, Minneapolis, MN, USA

**Keywords:** *In vitro* fermentation, *in vitro* intestinal models, animal dietary models

## Abstract

The gut microbiome is a critical mediator of human health. As the intestinal microbiota is far more metabolically diverse than humans, it plays a significant role in the digestion of food, particularly food components that are nutritionally inaccessible to the human host. While no system can fully recapitulate the *in vivo* interactions of food, the host, and the gut microbiome in the human body, *in vitro* and animal model tools are critical for studying these complex relationships. Here, we review many of the common *in vitro* and animal models used to manipulate and study how the gut microbiome affects and is affected by diet. We focus on colonic fermentation systems, with or without small intestinal contribution, bioreactors with both microbial and host epithelial cell components, and animal models that have been developed to study these relationships. We will review the limitations of these systems while also discussing new innovations that seek to address these limitations.

## Introduction

The human gastrointestinal tract harbors more microbial cells than human cells do in the body.^1^ This gut microbiome is markedly diverse, with several thousand distinct bacterial species,^2^^,^^3^ as well as viral, fungal, and archaeal components.^4-6^ Critically, the gut microbial metabolic potential is far broader than the enzymatic capability encoded by the human genome alone,^7^ allowing for the breakdown of food components that are indigestible to the host and the production of novel metabolites.^8^^,^^9^ Understanding interactions between food and intestinal microbes is therefore vital to investigate how the interplay between these compartments affects human health. However, studying this in human subjects can be difficult owing to human genetic variability, sampling challenges in a living person, and difficulty in normalizing diet across a study.

### Challenges in characterizing *in vivo* human diet‒microbiome interactions

Understanding diet‒microbiome interactions requires an understanding of the diversity and regionalization of the human gut microbiome. While fecal samples are most often used to investigate human microbial communities, we know that the diversity and function of these communities change across the gastrointestinal (GI) tract.^10^^,^^11^ These changes are thought to be related to host factors, including pH, bile acid content, oxygenation, and GI transit time. As a result, human gut microbial communities differ across different GI segments. For example, the human duodenum has a relatively low microbial burden of around 10^1^–10^3^ colony-forming units per milliliter (CFU/mL) and populations dominated by *Bacillota* and *Proteobacteria* phyla.^12^^,^^13^ However, the fecal microbiome is dense (10^10^–10^12^ CFU/mL) and much more diverse, dominated by the phyla *Bacteroidota* and *Bacillota* with substantially lower abundance of *Proteobacteria.*^12^^,^^13^ In addition, microbial communities in the intestinal lumen differ from those associated with the mucosa, likely due to local factors such as increased oxygen levels.^11^ As a result, diet–microbiome interactions will likely vary depending on the region of the GI tract that is assessed.

Many studies rely on fecal samples as a proxy for human intestinal microbiota. One benefit of fecal samples is the ease of collection, enabling in-depth sampling over time from the same individuals.^14^ These samples likely provide a good representation of the diversity and function of microbial communities from the human colonic lumen.^15^ However, they are less likely to accurately capture more proximal intestinal communities or mucus-associated communities. Furthermore, they can be rapidly altered by dietary changes or antibiotic use.^16^^,^^17^

Probing other regions of the GI tract is more challenging. Standard upper and lower endoscopies can obtain both luminal and mucosal samples from the stomach, duodenum, proximal jejunum, terminal ileum, and colon, but sampling of the deep small bowel is more limited. Jejunal aspirates have been classically used as a way to evaluate this region of the small intestine.^18^ Because these methods of sampling require intervention on human subjects, however, it is much more difficult to obtain longitudinal samples compared to fecal collection. In addition, the bowel preparation required for colonoscopy can alter microbial communities^19^ while a nil per os requirement for both upper and lower endoscopy limits *in vivo* evaluation of the effects of diet on the microbiome.^20^

While emerging tools seek to address some of these issues,^21^ there remains a strong need for *in vitro* and animal models to dissect microbial relationships in the GI tract, including diet‒microbiome interactions. Although no model system can fully capture the complexity of human, food, and microbial interactions, several *in vitro* and animal models have been developed to explore these relationships while allowing for a level of control and sampling that is infeasible in humans. In this review, we introduce many of these model systems and discuss their relative strengths and weaknesses for mimicking interactions in the human gut.

## *In vitro* models

**Figure 1.**
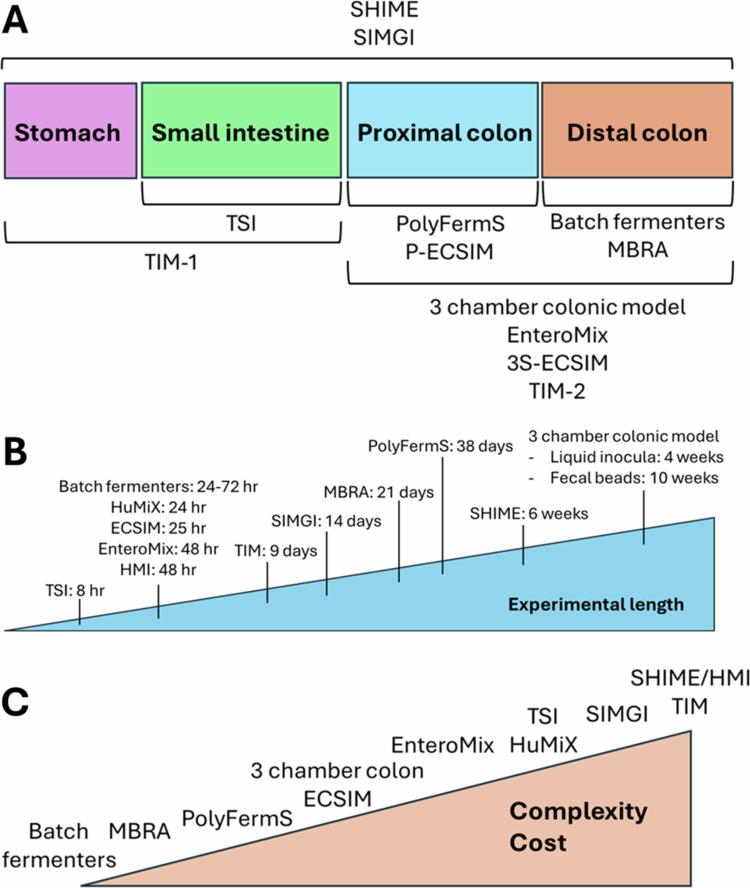
Comparison of *in vitro* models of diet–microbiome interactions. (A) Different *in vitro* models have been designed to mimic distinct components of the human GI tract. (B) Comparison of the experimental lengths used in different *in vitro* models. (C) Relative complexity and cost of different *in vitro* models of the human GI tract.

Broadly speaking, *in vitro* models of diet‒microbiome interactions manipulate the characteristics of fermentation chambers, such as pH, temperature, and mixing, to simulate the human GI tract (Figure 1). Inoculation of these systems with the human intestinal microbiota and nutritive components allows for carefully controlled analysis of the metabolic outputs of gut microbial digestion (Table 1). These models have differing benefits and drawbacks, including representation of distinct portions of the human GI tract (Figure 1A), variability in experimental length (Figure 1B), and differences in model complexity and cost (Figure 1C). More recent models incorporate human cells or tissue to add input from host cell crosstalk (Table 2).

**Table 1.** Comparison of *in vitro* fermentation models of food–microbiome interactions.

Model	Advantages	Disadvantages	Typical uses	Accuracy	Reference
Batch fermenters	Easy to set up	Time limited	Dietary fiber metabolism	Limited	Mikkelsen et al.^22^
	Useful for high-throughput screening	Minimal environmental control	Rapid metabolic screening		Lesmes et al.^23^
		No incorporation of input or waste system	Xenobiotic metabolism		Gietl et al.^24^
		No host modeling			Shi et al.^25^
MBRA	Simple setup	Only models distal colon	*Clostridioides difficile* colonization resistance	Similar microbial community across replicates and compared to gnotobiotic mice colonized with same sample	Robinson et al.^26^
	Continuous fermentation	No host modeling			Auchtung et al.^27^
	Parallel comparison of multiple conditions				Duquesnoy and Chassaing^28^
Three-chamber colonic model	Continuous fermentation	No input from small intestine	Dietary fiber metabolism	Comparable community to that observed in colon of sudden death victims	Gibson et al.^29^
	Control of pH in three colonic chambers	No host modeling	Amino acid metabolism		Macfarlane et al.^30^
	Longer fermentation times		Complex food metabolism		Payne et al.^31^
			Microbial hydrogen utilization		
EnteroMix	Continuous fermentation	Shorter fermentation time	Dietary fiber/carbohydrate metabolism	Similar SCFA production compared to other colonic models	Makivuoko et al.^32^
	Small volume/size	No input from small intestine			
		No host modeling			
PolyFermS	Continuous fermentation	Only models proximal colon	Carbohydrate metabolism	Bacterial phyla present in ratios similar to those in human fecal samples	Zihler Berner et al.^33^
	Parallel comparison of multiple conditions	No host modeling	SCFA production		
ECSIM	Continuous fermentation	No input from small intestine	Carbohydrate metabolism	Lower microbial diversity compared to human fecal samples	Feria-Gervasio et al.^34^
	Modular options for single or three-chamber	No host modeling	SCFA production		
	Anaerobic environment maintained by bacterial gas production				
SHIME	Continuous fermentation	Lengthy setup	Dietary fiber metabolism	Close to human fecal diversity at steady state	Molly et al.^35^
	Incorporates digestion in upper GI tract	No peristaltic mechanism	Complex food metabolism		Grootaert et al.^36^
	Dialysis to mimic host nutrient absorption	No input of small intestinal microbes			Van Den Abbeele et al.^37^
					Duysburgh et al.^38^
TIM	Continuous fermentation	Complex system	Dietary fiber metabolism	Bacterial composition correlates well with human fecal composition and rat data	Minekus et al.^39^
	Incorporates digestion in upper GI tract	Lengthy setup	Xenobiotic metabolism		Minekus et al.^40^
	Dialysis to mimic host nutrient absorption	No input of small intestinal microbes	Probiotic survival		
	Peristaltic mechanism	Only passive nutrient absorption			
	Includes gastric chamber				
SIMGI	Continuous fermentation	No absorption of nutrients	Dietary fiber fermentation	Bacterial composition correlates well with human fecal composition	Barroso et al.^41^
	Gastric chamber with peristalsis	Lengthy setup	Complex food digestion		
	Use for up to 14 d	No input of small intestinal microbes			
	Simpler than SHIME or TIM				
TSI	Smaller volumes/size	No colonic portion	Probiotic survival	Synthetic small intestinal community based on existing data, post-experimental diversity not examined	Cieplak et al.^42^
	Includes small intestinal microbes				
	Dialysis to mimic host nutrient absorption				

**Table 2.** Comparison of *in vitro* host-microbe models for food–microbiome interactions.

Model	Advantages	Disadvantages	Reference
HMI	Uses bacteria from SHIME fermenter	Short (48 h) experimental length	Marzorati et al.^43^
	Benefits of SHIME fermentation (Table 1)	May not support obligate anaerobes	
	Human cell line (Caco-2)	Lacks non-epithelial human cells	
HuMiX	Supports anaerobic bacterial growth	Short (24 h) experimental length	Shah et al.^44^
	Human cell line (Caco-2)	Lacks non-epithelial human cells	
		Minimal testing with complex microbial communities	
Gut-on-a-chip	Longer experimental length (up to 7 d)	May not support obligate anaerobes	Kim et al.^45^
	Differentiation of multiple epithelial cell types from Caco-2 cells	Lacks non-epithelial human cells	
		Minimal testing with complex microbial communities	
Intestinal organoids	Derived from primary human cells	May not support obligate anaerobes	Williamson et al.^46^
	Novel techniques available to access luminal space	Lacks non-epithelial human cells	

### Batch fermentation

The simplest models of diet‒microbiome interactions are batch fermentation systems. These systems are typically single-chamber systems prepared under anaerobic conditions (and kept anaerobic by flushing with nitrogen), maintained at a constant temperature (usually 37 °C), and inoculated with fecal material or bacterial strains along with the food component being studied. These systems typically run for no longer than 72 h (and usually 24–48 h) because of the rapid progression of bacterial cultures to the stationary phase after nutrient depletion and metabolic end-product accumulation.^47^ These batch fermentation systems are commonly employed to study dietary fiber metabolism. This has included modeling bacterial degradation of arabinoxylan, β-glucans, and xyloglucan,^22^ production of short-chain fatty acids (SCFA) via the metabolism of resistant starch,^23^ and the metabolism of galactooligosaccharides (GOSs).^24^ Batch fermentation models are much more limited than even other fermentation systems since they have no method of continuous input or output, do not typically have a way to simulate peristalsis, and cannot replicate the physiological changes that occur along the GI tract. However, as these methods are relatively easy to set up, these can be useful for high-throughput screening of diet‒microbiome interactions.^47^^,^^48^ A powerful recent example is work using fecal-derived microbial communities to test the metabolism of and microbial community response to over 700 drugs, highlighting the benefit of bioreactor systems to perform rapid, broad studies.^25^

### Continuous fermentation models

More advanced *in vitro* models of diet‒microbiome interactions include multichamber continuous fermentation arrangements. These models incorporate multiple fermentation chambers to simulate different regions of the GI tract with mechanisms for continuous nutrient input and waste removal, along with control of pH, temperature, and oxygen level.^47^

#### Three-chamber colonic model

**Figure 2.**
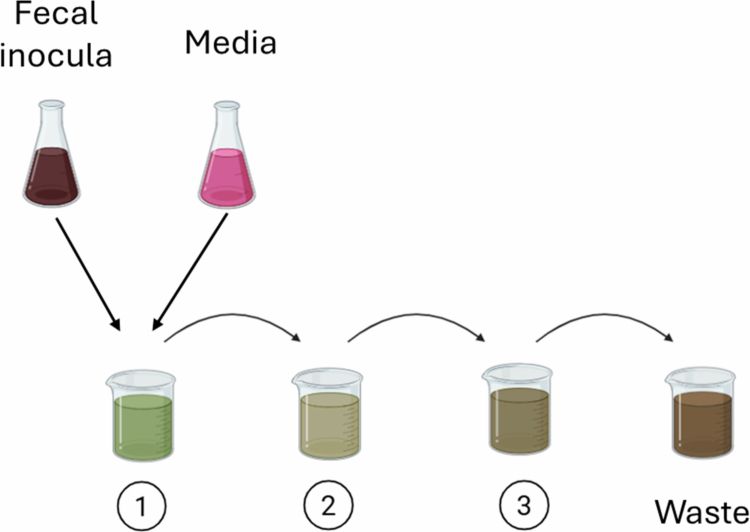
The classic 3-chamber colon model. Fecal material is used to seed the initial colon chamber (1) representing the proximal colon. Culture media is continuously fed into this chamber. Chambers representing the mid (2) and distal (3) colon are fed from the upstream chamber, and waste is removed from the final reactor.

The classic continuous fermentation system is a three-chamber system mimicking the human colon^29^ (Figure 2). In this system, a fecal inoculum is incubated with growth media and then serially added into three chambers with distinct pH values and volume to replicate the proximal, mid, and distal human colon. The overflow from the final chamber is removed as waste. These chambers are kept at 37 °C and continuously mixed. The three-chamber setup has been used to study microbial utilization of hydrogen for the metabolism of SCFAs, hydrogen sulfide, and methane,^29^ as well as amino acid metabolism.^30^ Notably, findings from microbial metabolism in each chamber have been found to correspond well to findings in the human colon via comparison to samples taken from sudden-death victims.^30^ Additional work has investigated the gut microbial metabolism of food using this system, food, including wheat dextrin^49^ and orange juice.^50^

The three-chamber fermentation system offers notable advantages over batch fermentation. Because there is continuous input of nutritive media and removal of waste, these fermenters can be used for much longer—up to 120 d, in the original description of the model.^29^ Later findings, however, suggest that the length of experiments using this model may be more limited with liquid fecal inocula owing to loss of less competitive bacteria during initial stabilization.^31^ One method to overcome this loss and provide continuous input of bacteria as well as nutrition is via immobilization of fecal samples in porous beads, which can be added to the first chamber at different time points.^51^ Compartmentalization into three chambers with distinct physicochemical properties also increases the physiological validity of this system compared to batch fermenters. However, this model is still only representative of distal GI tract (colonic) fermentation.

#### Other colonic models

A similar system, the EnteroMix system, uses four serial fermentation chambers to mimic fermentation across the colon.^32^ This system uses smaller volumes and can be more easily used to evaluate different conditions in parallel, but as a result, it can only evaluate short fermentation periods of 48 h.^32^ This system has been primarily used to study bacterial carbohydrate metabolism, including polydextrose^32^ and lactose.^52^

The polyfermentor intestinal model (PolyFermS) system mimics fermentation in the proximal colon.^33^ In this model, bacteria are incubated in an initial fermentation chamber with conditions mimicking the proximal colon. Next, samples from this chamber are used to inoculate multiple parallel chambers. As a result, the same bacterial inoculum can be used under different conditions, such as with the addition of different nutritional components, or under the same conditions across multiple replicates.

Another system that allows parallel analysis of multiple conditions with continuous flow is the microbioreactor array (MBRA).^26^^,^^27^ This system is a set of up to 48 replicate bioreactors with a constant rate of input of media and waste removal in a heated anaerobic chamber. This system has been used to study the ability of different fecal communities to resist *Clostridioides difficile* colonization^26^ and appears to be stable for up to 21 d across replicates and in comparison to gnotobiotic mice colonized with the same human fecal sample.^27^ An updated version of the MBRA incorporates mucus to allow examination of the mucus-adherent gut microbial community, which is not a standard component of other high-throughput intestinal models.^28^

The environmental control system for intestinal microbiota (ECSIM) is another model of the human colon.^34^ This model can be used as a single reactor system mimicking the proximal colon (P-ECSIM) or as a three-reactor system to mimic the proximal, transverse, and distal colon (3S-ECSIM). Similar to other colon model systems, the input rate, temperature, and pH are tightly controlled. One unique feature of ECSIM is that an anaerobic environment is maintained via microbially-produced gases rather than flushing with nitrogen.

While these systems offer increased flexibility in experimental design compared to the classic three-chamber fermenter, they have many of the same drawbacks. In particular, they only mimic metabolism occurring in the colon without input from the small intestine or upper GI tract.

#### Simulator of the human intestinal microbial ecosystem (SHIME)

**Figure 3.**
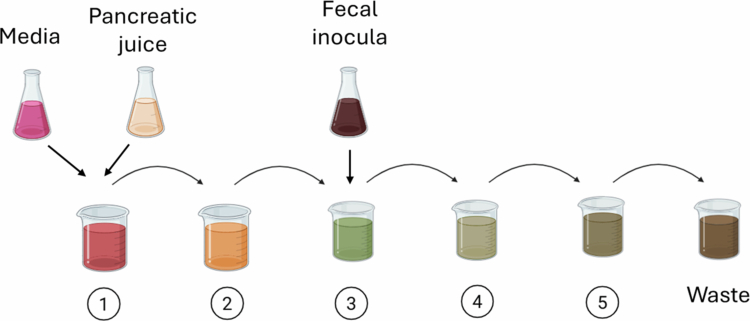
The SHIME model. Culture media and pancreatic juice are added to the first chamber (1), which represents the duodenum and jejunum. Next, effluent from each chamber is pumped to the next in the series, representing the ileum (2), proximal colon (3), mid colon (4), and distal colon (5). Fecal samples can be introduced into the proximal colon reactor.

The SHIME model combines lower GI tract fermentation systems like those described above with additional bioreactors simulating the small intestine^35^ (Figure 3). This system is composed of five chambers connected in series, representing the duodenum and jejunum, ileum, and colon (ascending, transverse, and descending). In addition, a separate chamber pumps pancreatic juice into the duodenal/jejunal chamber. As with the colonic simulators above, there is continuous flow into the system and out via waste. The reactor chambers for the small intestine also have a dialysis system to mimic host uptake of digested nutrients. Fecal samples can be inoculated into distal (colonic) chambers, similar to the three-chamber colon model described above. Variants on the SHIME model include Twin-SHIME, where two systems are run in parallel,^36^ mucus-SHIME, which adds mucin-coated beads for bacterial adherence,^37^ and Triple-SHIME, with three parallel systems including incorporation of a separate ileal compartment containing an ileal microbial consortium.^38^ The SHIME model has been used to study a variety of food and food component interactions with the gut microbiome. These include dietary fibers such as arabinoxylan, arabinogalactan, fructooligosaccharides (FOS), and inulin^35^^,^^36^^,^^53^^,^^54^ and more complex material like soybean powder, olive, and pomegranate.^55^^,^^56^

Although considered an excellent *in vitro* model of the human GI tract,^57^ there are some drawbacks to the SHIME system. In particular, this is a complex system requiring a lengthy setup period prior to use to allow microbial stabilization.^35^^,^^58^ The system is also expensive; one group has recently attempted to mimic SHIME using lower-cost materials to address this disadvantage.^59^ In addition, in SHIME, microbial communities in the colonic chambers may not precisely mimic those found in the human colon.^58^ As with the above models, mixing within each chamber is performed with magnetic stirring, which does not precisely replicate peristalsis and lacks any potential metabolic contribution from small intestinal microbes (other than in the newly developed Triple-SHIME system).

#### TNO intestinal model (TIM)

**Figure 4.**
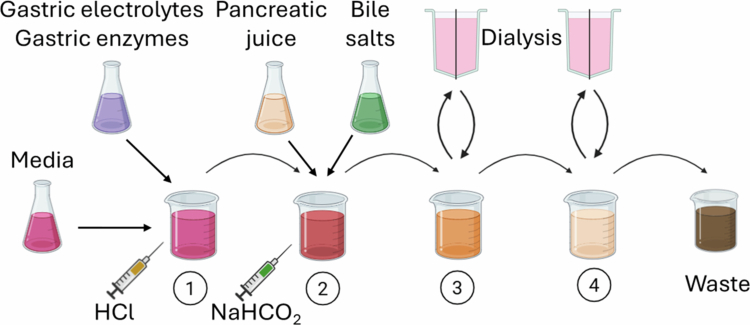
TIM-1 model. The four chambers simulate the stomach and small intestine. The gastric chamber (1) receives input from culture media as well as gastric electrolytes and enzymes. It is mixed via a flexible plastic enclosure to mimic peristalsis and pumped to the duodenal chamber (2), where pancreatic juice and bile salts are added. Both the gastric and duodenal chambers are under careful pH control, and either HCl, sodium bicarbonate, or water can be added to adjust as needed. The duodenal chamber is pumped into the jejunal (3), and ileal (4) chambers, both of which are connected to a dialysis system to mimic nutrient absorption.

The TIM model consists of two components: TIM-1 (Figure 4), a system of four bioreactors that mimic the stomach and small intestine,^39^ and TIM-2, a set of four chambers that replicate the colon.^40^ A major difference between TIM and the systems listed above is that there is a peristaltic mechanism to move fluid from one bioreactor to the next. In addition, dialysis membranes allow passive uptake of molecules of up to 5 kDa in the small intestine chambers and 50 kDa in the colon. The system is computer-controlled to maintain or adjust the peristaltic rate, pH, enzyme secretion, bile salt concentration, transit time, and absorption in each chamber, allowing for significant physiological control. The TIM system is commonly used for modeling the metabolism and uptake of xenobiotics such as pharmaceuticals^60^ as well as for studying the effects of fecal microbes on dietary fiber digestion.^40^

Similar to SHIME, the TIM system is considered a reliable model of the human GI tract.^47^ One study examined the metabolism of the dietary fibers arabinoxylan and inulin in both TIM-2 and SHIME and found that the effects on the microbial composition and production of SCFA were similar in both systems.^61^ Furthermore, these findings were similar to those of a trial of these two fibers in a rat model.^62^ However, like SHIME, the TIM system is complex and requires substantial setup. Although TIM has been used to study the survival of probiotic species and effects on the colonic microbiota,^63^^,^^64^ this model typically does not incorporate the small intestinal microbiota. In addition, although the use of dialysis membranes simulates passive absorption along the GI tract, active absorption and digestion via brush border enzymes from the host epithelium are lacking.

#### Simulator gastro-intestinal (SIMGI)

SIMGI is a system composed of five bioreactors designed to simulate the full human GI tract.^41^ The first chamber is composed of two rigid plastic segments with an inner flexible casing. Food and/or culture media are added to the inner casing. The addition or removal of water between the rigid outer layer and the flexible casing deforms the inner casing, mimicking gastric peristaltic mixing. The second SIMGI chamber models the small intestine, is fed from the gastric chamber, and, like the SHIME model, has pancreatic enzymes added. The three final chambers represent three segments of the colon. Like the TIM system, the individual compartments in SIMGI are monitored by a computer for maintenance of a constant pH within each chamber. A fecal inoculum is added to the colonic chambers and allowed to stabilize for two weeks, with similar transitional and final microbial compositions to SHIME.^62^ Following stabilization, dietary interventions can be applied for up to 14 d.^65^ The SIMGI model has been used to examine the effects of red wine,^66^ chia seed,^67^ and citrus pectin^65^ on human fecal microbial composition and SCFA production.

Although this model incorporates physiologic gastric peristalsis, an advantage over other systems, many of the same drawbacks apply. As with the above models, there is no incorporation of small intestinal microbes. In addition, although this system is easier to set up than TIM or SHIME, there is no simulation of host nutrient absorption.

#### The smallest intestine (TSI)

The TSI system exclusively models the small intestine with a focus on the use of smaller volumes.^42^ The system comprises the input of simulated nutrient media into a duodenal chamber, which is also inoculated with pancreatic juice and bile salts. In the jejunal chamber, dialysis membranes simulate host nutritional uptake. Finally, in the ileal chamber, a defined microbial population is added to simulate the distal small intestinal community. Like other newer models, pH, mixing time, and waste removal are monitored and maintained via computers. A variant of the model can incorporate simulated gastric juices.^42^ The small volumes of each chamber (12 mL) make this system somewhat easier to set up and maintain, and the use of small intestinal bacteria in the final chamber is an advantage compared to the above systems. However, as the TSI models only the small intestine, there is no colonic fermentation.

### Host cell‒microbial models

A critical drawback to all of the above systems is the lack of incorporation of host cells. Several models have been developed to include human-derived epithelial cells to overcome this deficiency. Most commonly, these systems rely on the Caco-2 human adenocarcinoma-derived colonic epithelial cell line, which mimics the expected properties of the colonic epithelium when grown in a monolayer.^68^ Other systems use alternative cell lines or *ex vivo* cultures of human or animal intestinal tissue.

### Host-microbiota interaction model (HMI)

**Figure 5.**
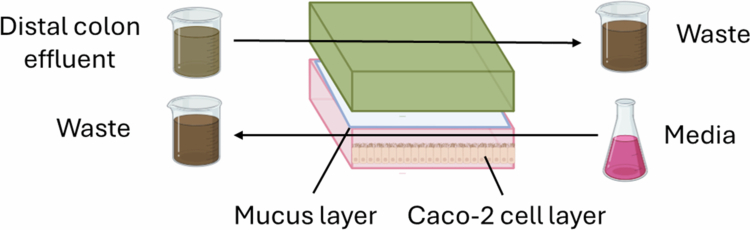
HMI model. This system consists of two chambers separated by a mucus layer. The top chamber receives outflow from a SHIME fermenter, which seeds it with bacteria. The bottom chamber is cultured with Caco-2 epithelial cells, which are fed by cell culture media. The mucus layer allows metabolite exchange between the bacterial and epithelial compartments while avoiding direct bacteria‒epithelium contact.

In the HMI system, Caco-2 and bacterial cells are cocultured in a chamber separated by a mucus layer^43^ (Figure 5). This mucus layer allows the diffusion of metabolites but prevents toxicity from the direct adherence of bacteria to epithelial cells. The bacterial layer is seeded by the outflow from a SHIME system with a countercurrent flow of cell culture media through the epithelial cell compartment, and the effluent is returned to the same SHIME reactor. The HMI system can be used to study epithelial–microbial interactions for up to 48 h, after which there is substantially decreased viability of the epithelial cells.

While 48 h is longer than other *in vitro* methods of modeling the microbial‒epithelial interface,^69^ this time frame is still much more limited than many of the bioreactors discussed above. Furthermore, while the incorporation of epithelial cells is a step closer to accurately modeling human intestine‒gut microbial interactions, the Caco-2 cell line may not perfectly mimic normal epithelial cells, and the HMI system lacks other integral cell types from the colonic mucosa, including secretory cells, immune cells, and fibroblasts.^70-72^

### Human-microbial crosstalk model (HuMiX)

**Figure 6.**
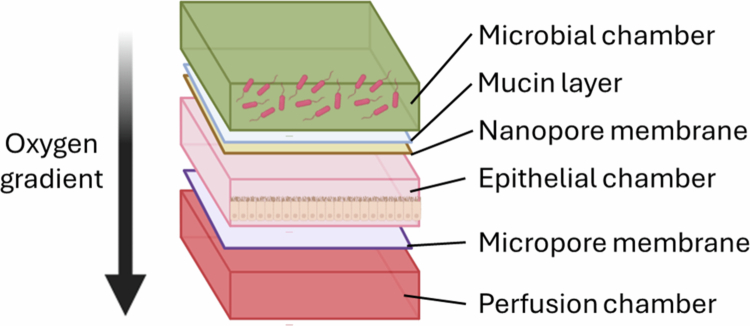
HuMiX model. This model consists of three stacked chambers (microbial, epithelial, and perfusion). The microbial and epithelial chambers are separated by a mucin layer and a nanoporous membrane. The epithelial chamber contains a layer of Caco-2 cells cultured on a microporous membrane coated with collagen, which is fed with nutritive media from the perfusion chamber. The oxygen sensors in the system confirm an increasing oxygen concentration from the microbial to the perfusion chambers, mimicking the gradient seen *in vivo*.

The HuMiX model is designed to examine human and microbial interactions via coculture under conditions mimicking those found in the *in vivo* GI tract^44^ (Figure 6). This system consists of three channels separated by porous membranes to allow communication between a microbial chamber, an epithelial cell chamber, and a culture media chamber. The membrane between the microbial and epithelial chambers is coated with mucin prior to seeding with a human epithelial cell line and inoculation with a bacterial strain. This model also includes oxygen sensors to track the dissolved oxygen concentration, which confirms that the final oxygen gradient from microbial to epithelial cell channels accurately mimics the gradient found *in vivo*. Using this model, the authors tracked epithelial transcriptional responses to coculture with *Lactobacillus rhamnosus* GG (LGG), a probiotic bacterial strain,^73^ and *Bacteroides caccae*, an obligate anaerobic member of the human gut microbiome.^74^ Notably, *B. caccae* was able to survive in this system, suggesting that the observed oxygen concentration in the microbial chamber was permissible for anaerobic bacterial growth. Subsequently, one publication has used the HuMiX system to study interactions between prebiotic fiber, LGG, and Caco-2 cells, and determined that the combination of fiber and LGG decreased the expression of genes with oncogenic potential in Caco-2 cells.^75^

The HuMiX model therefore offers an advantage over HMI in providing an environment suitable for anaerobic bacterial growth at the epithelial interface. However, compared to HMI, the coculture time is shorter (24 h), potentially limiting the data that can be obtained. Furthermore, this model was only tested with a very limited bacterial community. In addition, non-epithelial cell components from the host are still lacking in this model.

### Gut-on-a-chip

Gut-on-a-chip models can be used as microfluidic models of host cell–microbial interactions. Over the past decade, several models have emerged to study these interactions as well as host physiology and gut permeability, epithelial–immune crosstalk, and disease pathophysiology. A recent review of these models extensively describes their properties and the benefits and drawbacks of using these systems to study the human intestinal epithelium.^76^ For the purposes of this review, we will focus on the first gut-on-a-chip model as well as the limited data available from these models to examine diet‒microbiome interactions.

**Figure 7.**
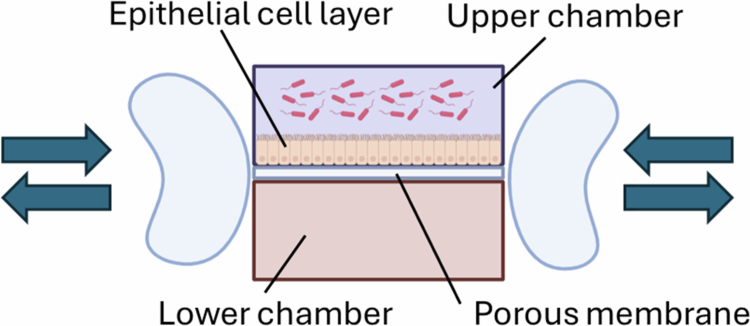
Gut-on-a-chip model. This model consists of two chambers (upper and lower) supplied with a continuous flow of culture media, separated by a porous membrane seeded with Caco-2 cells. Vacuum chambers lateral to the device provide mechanical deformation, which mimics peristalsis. The upper chamber can be inoculated with bacteria.

The earliest described gut-on-a-chip system consists of two fluid channels separated by a porous membrane, which is seeded with Caco-2 cells^45^ (Figure 7). The vacuum chambers on the side of the channels can stretch the channels, providing mechanical stress to the cells. Critically, peristaltic flow of culture media over the cells induces them to develop villus-like structures, resulting in formations more similar to human colonic epithelium than a flat layer of cells.^77^ In addition, the cells differentiate into Paneth cells, enteroendocrine cells, and goblet cells, and produce their own mucus layer.^77^ In the original description of this model, the authors cocultured LGG with the epithelial cells, allowing examination of the effects of this bacterial strain on colonic epithelial physiology.^45^

The dynamic assembly of an epithelial layer similar to *in vivo* tissue is a large advantage to this system. In addition, experiments for up to a week are possible. However, there are few to no data using this model to study diet‒microbiome interactions, and even the published work with a single probiotic strain is very different from examining a complex microbial community such as the gut microbiome. This model is microaerophilic, so it may not be appropriate or feasible to use to study fecal microbial metabolism. In addition, host immune elements are lacking in this model.

In subsequent gut-on-a-chip models, many of these aspects have been addressed, including the use of human stool communities and the addition of immune and endothelial cells.^76^^,^^78^^,^^79^ However, work examining diet‒microbiome interactions is rare. One recent paper studied interactions between fecal microbial communities and dietary fiber (inulin)^80^ using a newly developed intestinal explant on a chip model.^81^ The intestinal explant model allows for *ex vivo* culture of tissue for up to 24 h. The authors incubated the stool samples with or without inulin and then treated the intestinal explants with the supernatant from the stool cultures. They examined the effects of inulin supplementation on SCFA production by fecal communities and, in turn, whether this affected epithelial viability and barrier integrity. However, the temporal and physical separation of bacterial fermentation and host tissue is a substantial drawback of this work, particularly as other gut-on-a-chip systems have directly co-cultured fecal samples with human cells.^78^^,^^82^ Nonetheless, these systems have unexplored potential for evaluating diet‒microbiome interactions.

### Intestinal organoids

Intestinal organoids are three-dimensional structures derived from isolated intestinal crypts or intestinal stem cells.^83^ These organoids form an epithelial layer surrounding an inner lumen and mimic *in vivo* gut epithelium, with appropriate cell differentiation and polarization.^83^ However, because the assembly of an organoid results in a sphere, there is no access to the inner space, which is equivalent to the luminal compartment where food and microbes would reside *in vivo*. A recent study, however, used microinjections to insert fecal bacteria into the luminal space of intestinal organoids and observe growth for up to 96 h afterward.^46^ This organoid microinjection system was later used to study the effects of prebiotic GOS and the gut microbiome, with a focus on the aging gut.^84^ This study used mouse-derived colonoids injected with mouse fecal microbial communities from young or old mice, with or without GOS, and showed that the bacterial composition of the resulting cultures was significantly different across these groups.

Further work with this model could represent a novel way to study interactions among food, human-derived intestinal organoids, and complex communities of intestinal bacteria. However, the oxygen levels needed to sustain organoid viability may not be compatible with many anaerobic bacteria. In addition, there are still critical elements missing from the host, such as immune cells.

#### Limitations of *in vitro* models

While the above models provide a range of options for pursuing studies of human diet‒microbiome interactions, they all continue to have some limitations that cannot fully reproduce human physiology. For example, none of these models incorporate host bile acid metabolism and recycling. Bile acid metabolism by gut microbes and uptake by the host in the distal small intestine or colon, with further modifications by the liver, has profound impacts on digestive, immune, and microbiology physiology.^85-87^ These models also lack incorporation of the enteric nervous system (ENS). Beyond controlling peristalsis, the ENS has critical roles in nutrient absorption and immune regulation^88^ and can respond to microbial metabolites such as SCFAs and tryptophan derivatives.^89^^,^^90^

In addition, while newer *in vitro* models tackle many of the issues inherent in older technologies, such as incorporation of host epithelial cells and even immune cell populations, there is some loss of beneficial aspects of these older models, such as simulated host digestion of complex foods. Therefore, animal models remain a critical method for investigating diet‒microbiome interactions (Table 3).

**Table 3.** Comparison of animal models for food–microbiome interactions.

Model	Advantages	Disadvantages	References
Mouse	Grossly similar anatomy	Higher gastric pH	Mestas and Hughes^92^
	Cheaper husbandry costs	Non-glandular portion of the stomach	Nguyen et al.^93^
	Breed quickly	Coprophagic	Hugenholtz et al.^94^
	Similar microbiome (phylum level)	Cecal fermentation	Kararli^95^
	Gnotobiotics widely available		Gibbons and Spencer^107^
			Xiao et al.^109^
			Turnbaugh et al.^91^
Pig	Similar anatomy and gastric physiology	Cecal fermentation	Graham and Aman^97^
	Fecal microbiome similar to humans	More costly	Heinritz et al.^98^
	Gnotobiotics available		Leser et al.^99^
			Liu et al.^100^
			Miniats and Jol^101^
Nonhuman primate	Evolutionarily similar to humans	Differences in anatomy and physiology are likely driven by diet	Hatton et al.^102^
	Similar anatomy and gastric physiology	Differential microbial response to diet changes	McKenna et al.^103^
	Similar microbiome (phylum level)	Expensive husbandry	Eichberg et al.^104^
	Useful for long-term studies (years)	Very limited gnotobiotic availability	
Others (Drosophila, zebrafish)	Cheaper husbandry costs	Less similar to humans	Douglas^105^
	High reproductive rates		Anon et al., 2017^106^
	Gnotobiotics available		

## Animal models

### Mouse models

Mice are a commonly used model for many aspects of diet‒microbiome‒host interactions, particularly for studies involving the immune system^92^ and/or microbial contributions to health and disease.^93^ Mice offer several advantages as a model system, including relative similarity to humans in GI physiology and anatomy, rapid reproductive times, small size, and ease of housing and husbandry.^47^^,^^48^^,^^93^ However, there are important differences in food digestion and fermentation, microbial communities, and host immune systems that are critical to consider in the use of these models.

The GI tract of mice is superficially similar to that of humans. For example, mice and humans have similar digestive organs, and the ratio of the intestinal surface area to the body area is comparable in both species.^93^ However, there are several important differences exist that can affect GI microbial populations. For example, mice, but not humans, have a non-glandular forestomach, which is covered in biofilms composed of *Lactobacillus* species.^94^ In addition, mice have an enlarged cecum, which acts as a site of substantial microbial fermentation, whereas no equivalent organ exists in humans.^93^ Mice also have a much faster GI transit time and a higher gastric pH,^95^ which likely impacts both food digestion as well as microbial survival.

The intestinal immune systems of humans and mice, while superficially similar, also display some key differences.^92^^,^^107^ For example, murine Paneth cells, an epithelial cell type critical for innate immune defenses in the gut, secrete more than twenty forms of antimicrobial peptide defensins, whereas human Paneth cells produce only two.^107^ In addition, Peyer's patches, which are important sites of adaptive immune (B and T cell) induction, are substantially more concentrated in the distal ileum of humans compared to mice.^108^

The composition of distal GI bacterial communities in mice and humans is similar on a phylum level, both being dominated by *Bacillota*, *Bacteroidota*, and *Proteobacteria* phyla.^96^ However, 85% of 16S rRNA sequences found in mice represent bacterial genera that have not been found in humans.^96^ Furthermore, by examining shotgun metagenomics sequences rather than 16S rRNA sequences, only 4% of genes in mice were shared in human samples.^109^ Nonetheless, over 95% of functional groups of genes were shared between mice and humans, suggesting that while exact bacterial taxa may differ, the metabolic capacity of microbes from both species has high overlap.^109^

Germ-free mice offer a powerful tool to study the contribution of gut microbes to host physiology.^110^ In addition to mice lacking any microbial community, germ-free mice can be inoculated with individual strains of bacteria, fecal samples from other mice or humans, or even complex, defined synthetic microbial communities to create gnotobiotic organisms.^110^^,^^111^ In a landmark paper, germ-free mice were colonized with human fecal microbial communities and fed either a standard mouse chow or switched to a “Western” high-fat/high-sugar diet.^91^ The diet change rapidly induced shifts in the gut microbial community, was associated with increased adiposity, and could drive obesity in mice given a fecal transplant from mice with this altered microbial community. This work clearly demonstrated that diet not only drives changes in the gut microbial composition but also that these changes can affect host health.

Differences also exist between mouse and human small intestinal microbial communities. Mice are coprophagic, which probably affects the composition of these communities.^112^ Sampling of the human small intestinal microbiota has been challenging and typically involves sampling from patients with ileostomies,^113^ but newer technologies are emerging to characterize the microbial composition of this site.^21^ Emerging work uses the results of this human small intestinal sampling to design novel, defined communities for use in gnotobiotic mice and investigates small intestinal microbial responses to a fiber-deficient diet *in vivo.*^114^

### Pig models

Another *in vivo* model used for diet–microbiome interactions is pigs, which have historically been used as a model of dietary fiber digestion.^97^ Compared to rodents, pigs are more anatomically and physiologically similar to humans.^98^ For example, GI transit time is similar in pigs and humans.^97^ However, there are some notable differences, including a much greater capacity for hindgut fermentation of fiber^97^ and a much longer GI tract in pigs.^98^

As in mice, the gut microbial composition of pigs is similar to humans at the phylum level, dominated by *Bacteroidota* and *Bacillota.*^99^ A very recent paper deeply characterized the pig fecal microbiome via shotgun sequencing and metagenome-assembled bacterial genomes (MAGs).^100^ Comparison of MAGs obtained from pigs in this study to available sequencing data for humans, mice, sheep, and cattle found that the greatest similarity was between humans and pigs. Therefore, pigs may offer an improved model over mice to study the human microbiome and, potentially, diet‒microbiome interactions.

Indeed, a number of older studies have examined the effects of prebiotic fibers such as GOS, FOS, or inulin on pig microbial composition and SCFAs. For example, one group evaluated the effect of inulin on microbial composition, and found that *Bifidobacteria* and *Lactobacilli* were increased, with a concomitant decrease in *Clostridia* and *Enterobacteriaceae* taxa.^115^ Another group used a suckling pig model to investigate whether polydextrose was suitable as a substitute for human milk oligosaccharides in infant formula, which is known to increase the abundance of *Bifidobacteria* species in the infant gut.^116^ However, they found that there was little impact on *Bifidobacteria*, and instead of an increase in *Lactobacilli* and propionic acid production.

Like mice, gnotobiotic pigs provide an opportunity to study defined microbial communities in the context of food and digestion.^101^ For example, a recent paper evaluated changes in gut microbial communities from malnourished children when inoculated into gnotobiotic pigs.^117^ The authors found that the microbial metabolic characteristics found in malnourished compared to healthy children, such as an increase in the abundance of genes associated with carbohydrate biosynthesis, were recapitulated in the pigs. Other work has used gnotobiotic pigs colonized with transplanted human fecal bacteria to understand the effects of malnutrition on vaccine efficacy.^118^

The similarities in anatomy, digestive physiology, and microbial profiles to humans may make pigs an improved option over mice for studying food‒microbe interactions. However, they are more costly and associated with greater husbandry challenges owing to their size and slower reproductive rates. As mentioned above, the differences in fermentation between pigs and humans could still limit their utility, particularly for studying distal GI carbohydrate metabolism.

### Nonhuman primate models

Nonhuman primates (NHP) are the closest evolutionary model to humans. On a gross level, the GI anatomy of the NHP is reasonably similar to that of human anatomy; for example, both have a simple glandular stomach, unlike mice.^102^ However, there are distinctions in anatomy across primate species that are linked to differences in diet.^102^ In terms of physiology, the fasted pH of the stomach in the NHP is highly variable but overall slightly higher than that in humans, and while NHP experiences a similar rise in pH following a meal, this increase lasts longer than that in humans.^102^

These anatomical and dietary differences are reflected in the gut microbial composition of the NHP. Macaques, for example, have distinct gut microbial profiles compared to both humans and mice, although all three species have similar dominant bacterial phyla.^103^ Nonetheless, the longevity of NHP compared to rodents offers the opportunity to study the long-term effects of diet. For example, one group used the NHP to evaluate the effects of a standard “Western” versus Mediterranean diet on the gut microbiome over a 2.5-y period.^119^ A second group later found that while macaque and human fecal microbial communities undergo similar shifts to a Mediterranean diet, responses to the “Western” diet were divergent.^120^ Overall, this suggests that the NHP may be useful for studying diet‒microbiome changes in some contexts but does not completely replicate findings from human studies.

Models of gnotobiotic NHP have been described,^104^ but these are quite costly and require highly specialized husbandry conditions that are less widely available than those for mice or pigs. These costs, combined with evidence of the differential response of the gut microbiome to diet in NHP versus humans, make gnotobiotic NHP less appealing as a model system.

### Other animal models

Other animal model systems are less similar to humans than those mentioned above. However, some of these, such as zebrafish and *Drosophila*, are simpler to use and have gnotobiotic options available.^105^^,^^106^ For example, one group has used gnotobiotic zebrafish to study the effects of the gut microbiome on fatty acid metabolism and absorption.^121^ Despite their anatomical and physiological differences from humans, the low cost and ease of use of these animal models may be particularly useful when large-scale screening tools are desired.

## Future directions

Overall, many *in vitro* and animal model systems have been used for studying diet‒microbiome interactions. While this has uncovered critical insights into how intestinal microbes modulate and are modulated by dietary components, some caution is needed in extrapolating these findings to *in vivo* human conditions. A re-assessment of microbial or physiological accuracy for many of these models may be necessary as we gain updated knowledge about human digestive and gut microbial physiology. Emerging insights into microbial metabolism of food,^122^ bacterial bile acid modifications,^87^ or host responses to food,^123^ for example, may uncover previously unknown inaccuracies in these modeling systems.

There are many possibilities for improving these models to better replicate findings in humans. The inclusion of contributions from small intestinal microbes, particularly with *in vitro* models, has been lacking. As above, tools are emerging to better understand the human small intestinal microbiome and to use this knowledge to develop improved mouse models.^21^^,^^114^ These findings could also be applied to advanced *in vitro* models of the human digestive tract, such as the SHIME model, to investigate how dietary metabolism and absorption are impacted by small intestinal resident microbes.

Another novel direction could be to take advantage of the array of “humanized” mice, which are genetically modified to more closely resemble human physiology. For example, immunodeficient mice can be engrafted with human hematopoietic stem cells to generate mice with a humanized immune system.^124^ Such mice could be used to understand how the immune system is affected by the microbial metabolism of dietary components. As another example, researchers have developed a mouse model replacing murine cytochrome P450 expression in the intestinal epithelium with that of human cytochromes.^125^ This model could be used to understand how diet‒microbiome interactions are impacted by humanized intestinal metabolism. Such “humanized” mice could also be combined with gnotobiotic research, allowing the study of the interaction among the host, microbiome, and diet in a model system that mimics both human physiology and microbial composition.

While no model system perfectly replicates the human organism, current models for human diet‒microbiome interactions have matured immensely from simple batch fermenters. As we uncover more about how diet and the gut microbiome intersect via observational studies in humans, our model systems will be upgraded to better manipulate these interactions and uncover their effects on human health.

## Data Availability

Not applicable.
